# Supplementary material for: Sequential Transcriptome Changes in the Penumbra after Ischemic Stroke
Source: Int J Mol Sci. 2019 Dec 16;20(24):6349. doi: 10.3390/ijms20246349 (PMC6940916; doi:10.3390/ijms20246349)
Supplement: Supplementary file 1 [file ijms-20-06349-s001.zip › Table S1.pdf]

Top 10 Genes in the Protein–Protein Interaction (PPI) Network of Biological Processes in Gene Ontology Analysis

1 week			4 weeks			8 weeks		
**Brain development (total 234 genes)**			**Positive regulation of cell population proliferation (total 57 genes)**			**Negative regulation of multicellular organismal process (total 73 genes)**		
gene_symbol	description	Fc	gene_symbol	description	Fc	gene_symbol	description	Fc
Tp53	tumor protein p53	2.754	Igf1	insulin-like growth factor 1	2.352	Cd44	CD44 molecule (Indian blood group)	6.132
Casp3	caspase 3	2.403	Ptprc	protein tyrosine phosphatase, receptor type, C	2.640	Timp1	TIMP metallopeptidase inhibitor 1	6.297
Rhoa	ras homolog family member A	2.253	Timp1	TIMP metallopeptidase inhibitor 1	7.381	Ccl2	C-C motif chemokine ligand 2	2.657
Gnai1	G protein subunit alpha i1	-2.844	Cxcl12	C-X-C motif chemokine ligand 12	2.135	Tgfb1	transforming growth factor, beta 1	2.045
Syt1	synaptotagmin 1	-20.346	Mmp2	matrix metallopeptidase 2	2.172	Spp1	secreted phosphoprotein 1	9.088
Grin2a	glutamate ionotropic receptor NMDA type subunit 2A	-3.675	Tgfb1	transforming growth factor, beta 1	2.095	Col3a1	collagen type III alpha 1 chain	13.728
Ccr5	chemokine (C-C motif) receptor 5	3.140	Lgals3	lectin, galactoside-binding, soluble, 3	36.529	Vim	vimentin	10.175
Myc	myelocytomatosis oncogene	5.493	Rac2	ras-related C3 botulinum toxin substrate 2 (rho family, small GTP binding protein Rac2)	2.589	Dcn	decorin	6.114
Aif1	allograft inflammatory factor 1	10.359	Hmox1	heme oxygenase 1	2.392	Cd74	CD74 molecule	15.205
Gnao1	G protein subunit alpha o1	-5.734	Igf2	insulin-like growth factor 2	25.058	Anxa1	annexin A1	10.742
**Memory (total 76 genes)**			**Wound healing (total 32 genes)**			**Response to estradiol (total 27 genes)**		
gene_symbol	description	Fc	gene_symbol	description	Fc	gene_symbol	description	Fc

Bdnf	brain-derived neurotrophic factor	-3.076	Timp1	TIMP metallopeptidase inhibitor 1	7.381	Ccl2	C-C motif chemokine ligand 2	2.657
Gria1	glutamate ionotropic receptor AMPA type subunit 1	-4.583	Igf1	insulin-like growth factor 1	2.352	Col1a1	collagen, type I, alpha 1	13.868
Grin2b	glutamate ionotropic receptor NMDA type subunit 2B	-2.394	Sparc	secreted protein acidic and cysteine rich	2.122	Igf2	insulin-like growth factor 2	28.159
Grin2a	glutamate ionotropic receptor NMDA type subunit 2A	-3.675	Tgfb1	transforming growth factor, beta 1	2.095	Tgfb1	transforming growth factor, beta 1	2.045
Slc17a7	solute carrier family 17 member 7	-134.524	Col1a1	collagen, type I, alpha 1	15.053	Vim	vimentin	10.175
Cnr1	cannabinoid receptor 1 (brain)	-3.649	Dcn	decorin	4.806	Nqo1	NAD(P)H quinone dehydrogenase 1	2.160
Egr1	early growth response 1	-3.205	Cd44	CD44 molecule (Indian blood group)	7.931	Anxa1	annexin A1	10.742
Htr2a	5-hydroxytryptamine receptor 2A	-3.237	Igf2	insulin-like growth factor 2	25.058	Igfbp3	insulin-like growth factor binding protein 3	5.342
Snap25	synaptosomal-associated protein 25	-226.767	Col3a1	collagen type III alpha 1 chain	18.130	Cd4	Cd4 molecule	2.380
Cck	cholecystokinin	-98.243	Pf4	platelet factor 4	2.857	Pdgfra	platelet derived growth factor receptor alpha	2.334
**Cellular response to drug (total 140 genes)**			**Response to estradiol (total 27 genes)**			**Cellular response to lipid (total 43 genes)**		
gene_symbol	description	Fc	gene_symbol	description	Fc	gene_symbol	description	Fc
Tp53	tumor protein p53	2.754	Igf1	insulin-like growth factor 1	2.352	Ccl2	C-C motif chemokine ligand 2	2.657
Mapk8	mitogen-activated protein kinase 8	-2.178	Col1a1	collagen, type I, alpha 1	15.053	Icam1	intercellular adhesion molecule 1	2.102
Ccl2	C-C motif chemokine ligand 2	22.318	Igf2	insulin-like growth factor 2	25.058	Cd40	CD40 molecule	2.275
Myc	myelocytomatosis oncogene	5.493	Mmp2	matrix metallopeptidase 2	2.172	Cxcl13	C-X-C motif chemokine ligand 13	2.306
Icam1	intercellular adhesion molecule 1	6.542	Tgfb1	transforming growth factor, beta 1	2.095	Tgfb1	transforming growth factor, beta 1	2.045
Igf1	insulin-like growth factor 1	52.188	Vim	vimentin	13.221	Il18	interleukin 18	2.361

Hmox1	heme oxygenase 1	52.116	Bmp7	bone morphogenetic protein 7	5.416	Spp1	secreted phosphoprotein 1	9.088
Rhoa	ras homolog family member A	2.253	Igfbp3	insulin-like growth factor binding protein 3	2.123	Col1a1	collagen, type I, alpha 1	13.868
Il18	interleukin 18	10.222	Pdgfra	platelet derived growth factor receptor alpha	2.655	Cxcl16	C-X-C motif chemokine ligand 16	6.413
Gnai2	G protein subunit alpha i2	2.307	Igfbp2	insulin-like growth factor binding protein 2	14.026	Pf4	platelet factor 4	3.404
**Positive regulation of apoptotic process (total 181 genes)**			**Aging (total 34 genes)**			**Positive regulation of cell migration (total 39 genes)**		
gene_symbol	description	Fc	gene_symbol	description	Fc	gene_symbol	description	Fc
Tp53	tumor protein p53	2.754	Igf1	insulin-like growth factor 1	2.352	Cxcl12	C-X-C motif chemokine ligand 12	2.445
Casp3	caspase 3	2.403	Tgfb1	transforming growth factor, beta 1	2.095	Ccl2	C-C motif chemokine ligand 2	2.657
Mapk8	mitogen-activated protein kinase 8	-2.178	Timp1	TIMP metallopeptidase inhibitor 1	7.381	Icam1	intercellular adhesion molecule 1	2.102
Myc	myelocytomatosis oncogene	5.493	Mmp2	matrix metallopeptidase 2	2.172	Cxcl13	C-X-C motif chemokine ligand 13	2.306
Tlr4	toll-like receptor 4	3.459	Fos	FBJ osteosarcoma oncogene	-2.657	Ccl19	C-C motif chemokine ligand 19	3.688
Ccl2	C-C motif chemokine ligand 2	22.318	Clu	clusterin	2.194	Col1a1	collagen, type I, alpha 1	13.868
Mmp9	matrix metallopeptidase 9	17.343	Dcn	decorin	4.806	Anxa1	annexin A1	10.742
Casp8	caspase 8	3.386	Serping1	serpin family G member 1	21.823	Cd40	CD40 molecule	2.275
Agt	angiotensinogen	-18.171	Vim	vimentin	13.221	Il18	interleukin 18	2.361
Anxa5	annexin A5	2.050	Ucp2	uncoupling protein 2	2.745	Aif1	allograft inflammatory factor 1	2.155
**Regulation of synaptic vesicle exocytosis (total 59 genes)**			**Response to drug (total 64 genes)**			**Cell adhesion (total 44 genes)**		
gene_symbol	description	Fc	gene_symbol	description	Fc	gene_symbol	description	Fc
Syt1	synaptotagmin 1	-20.346	Mmp2	matrix metallopeptidase 2	2.172	Cd44	CD44 molecule (Indian blood group)	6.132
Stxbp1	syntaxin binding protein 1	-7.151	Igf1	insulin-like growth factor 1	2.352	Spp1	secreted phosphoprotein 1	9.088
Stx1a	syntaxin 1A	-23.647	Col1a1	collagen, type I, alpha 1	15.053	Cxcl12	C-X-C motif chemokine ligand 12	2.445

Rab3a	RAB3A, member RAS oncogene family	-23.394	Tgfb1	transforming growth factor, beta 1	2.095	Icam1	intercellular adhesion molecule 1	2.102
Cplx1	complexin 1	-62.030	Fos	FBJ osteosarcoma oncogene	-2.657	Col3a1	collagen type III alpha 1 chain	13.728
Syt2	synaptotagmin 2	-8.045	Hmox1	heme oxygenase 1	2.392	Lamb2	laminin subunit beta 2	2.149
Vamp1	vesicle-associated membrane protein 1	-14.025	Igf2	insulin-like growth factor 2	25.058	Itgb4	integrin subunit beta 4	10.949
Cplx2	complexin 2	-18.489	Sparc	secreted protein acidic and cysteine rich	2.122	Thbs2	thrombospondin 2	3.492
Syp	synaptophysin	-44.394	Vim	vimentin	13.221	Tgfbr2	transforming growth factor, beta receptor 2	2.195
Camk2a	calcium/calmodulin-dependent protein kinase II alpha	-139.806	Gpx1	glutathione peroxidase 1	2.167	Postn	periostin	-2.405
**Regulation of postsynaptic membrane potential (total 58 genes)**			**Ossification (total 24 genes)**			**Extracellular matrix organization (total 25 genes)**		
gene_symbol	description	Fc	gene_symbol	description	Fc	gene_symbol	description	Fc
Gria1	glutamate ionotropic receptor AMPA type subunit 1	-4.583	Igf1	insulin-like growth factor 1	2.352	Col1a1	collagen, type I, alpha 1	13.868
Grin2a	glutamate ionotropic receptor NMDA type subunit 2A	-3.675	Spp1	secreted phosphoprotein 1	10.971	Col3a1	collagen type III alpha 1 chain	13.728
Grm5	glutamate metabotropic receptor 5	-3.177	Mmp2	matrix metallopeptidase 2	2.172	Lum	lumican	2.924
Gabra1	gamma-aminobutyric acid type A receptor alpha1 subunit	-26.419	Col1a1	collagen, type I, alpha 1	15.053	Postn	periostin	-2.405
Gabrg2	gamma-aminobutyric acid type A receptor gamma 2 subunit	-12.334	Sparc	secreted protein acidic and cysteine rich	2.122	Fbln1	fibulin 1	6.400
Grin2b	glutamate ionotropic receptor NMDA type subunit 2B	-2.394	Igf2	insulin-like growth factor 2	25.058	Mmp14	matrix metallopeptidase 14	6.884
Gabrb2	gamma-aminobutyric acid type A receptor beta 2 subunit	-6.061	Tgfb1	transforming growth factor, beta 1	2.095	Fmod	fibromodulin	7.465
Gria2	glutamate ionotropic receptor AMPA type subunit 2	-8.968	Igfbp5	insulin-like growth factor binding protein 5	2.649	Tgfb1	transforming growth factor, beta 1	2.045

Grm1	glutamate receptor, metabotropic 1	-2.475	Ctsk	cathepsin K	3.212	Lgals3	lectin, galactoside-binding, soluble, 3	21.687
Grik1	glutamate ionotropic receptor kainate type subunit 1	-3.151	Twist1	twist family bHLH transcription factor 1	2.436	Col18a1	collagen type XVIII alpha 1 chain	3.034
**locomotory behavior (total 91 genes)**			**Negative regulation of cell population proliferation (total 41 genes)**			**Aging (total 34 genes)**		
gene_symbol	description	Fc	gene_symbol	description	Fc	gene_symbol	description	Fc
Gad1	glutamate decarboxylase 1	-3.543	Igf1	insulin-like growth factor 1	2.352	Ccl2	C-C motif chemokine ligand 2	2.657
Scn1a	sodium voltage-gated channel alpha subunit 1	-9.277	Cd44	CD44 molecule (Indian blood group)	7.931	Tgfb1	transforming growth factor, beta 1	2.045
Snap25	synaptosomal-associated protein 25	-226.767	Tgfb1	transforming growth factor, beta 1	2.095	Timp1	TIMP metallopeptidase inhibitor 1	6.297
Ppp1r1b	protein phosphatase 1, regulatory (inhibitor) subunit 1B	-4.625	Sparc	secreted protein acidic and cysteine rich	2.122	Icam1	intercellular adhesion molecule 1	2.102
Adora2a	adenosine A2a receptor	-2.655	Tgfbr2	transforming growth factor, beta receptor 2	2.579	Clu	clusterin	2.292
Mapk8	mitogen-activated protein kinase 8	-2.178	Lgals3	lectin, galactoside-binding, soluble, 3	36.529	Gpx1	glutathione peroxidase 1	2.092
Gnao1	G protein subunit alpha o1	-5.734	Hmox1	heme oxygenase 1	2.392	Nqo1	NAD(P)H quinone dehydrogenase 1	2.160
Cacna1a	calcium voltage-gated channel subunit alpha1 A	-5.096	Bmp7	bone morphogenetic protein 7	5.416	Itgb2	integrin subunit beta 2	2.361
Grm5	glutamate metabotropic receptor 5	-3.177	Timp2	TIMP metallopeptidase inhibitor 2	2.291	Serping1	serpin family G member 1	16.626
Grm1	glutamate receptor, metabotropic 1	-2.475	Gpc3	glypican 3	4.840	Mbp	myelin basic protein	-3.010
**Aging (total 138 genes)**			**Cellular response to cytokine stimulus (total 42 genes)**			**Negative regulation of cell population proliferation (total 42 genes)**		
gene_symbol	description	Fc	gene_symbol	description	Fc	gene_symbol	description	Fc

Tp53	tumor protein p53	2.754	Cxcl12	C-X-C motif chemokine ligand 12	2.135	Cd44	CD44 molecule (Indian blood group)	6.132
Igf1	insulin-like growth factor 1	52.188	Cd44	CD44 molecule (Indian blood group)	7.931	Lgals3	lectin, galactoside-binding, soluble, 3	21.687
Stat3	signal transducer and activator of transcription 3	2.550	Mmp2	matrix metallopeptidase 2	2.172	Ccl2	C-C motif chemokine ligand 2	2.657
Agt	angiotensinogen	-18.171	Spp1	secreted phosphoprotein 1	10.971	Tgfb1	transforming growth factor, beta 1	2.045
Mmp9	matrix metallopeptidase 9	17.343	Col1a1	collagen, type I, alpha 1	15.053	Tgfbr2	transforming growth factor, beta receptor 2	2.195
Bdnf	brain-derived neurotrophic factor	-3.076	Ifitm1	interferon induced transmembrane protein 1	34.439	Aif1	allograft inflammatory factor 1	2.155
Icam1	intercellular adhesion molecule 1	6.542	Cd74	CD74 molecule	10.829	Fcgr2b	Fc fragment of IgG, low affinity IIb, receptor	2.119
Ccl2	C-C motif chemokine ligand 2	22.318	Ifitm3	interferon induced transmembrane protein 3	4.490	Gpc3	glypican 3	3.974
Fos	FBJ osteosarcoma oncogene	-2.452	Gbp2	guanylate binding protein 2	4.468	Tspo	translocator protein	2.792
Hras	Harvey rat sarcoma virus oncogene	-2.113	Cyba	cytochrome b-245 alpha chain	4.201	Inhba	inhibin beta A subunit	-2.184
**Regulation of cation channel activity (total 73 genes)**			**Response to lipopopysaccharide (total 30 genes)**			**Inflammatory response (total 31 genes)**		
gene_symbol	description	Fc	gene_symbol	description	Fc	gene_symbol	description	Fc
Dlg4	discs large MAGUK scaffold protein 4	-8.413	Igf1	insulin-like growth factor 1	2.352	Ccl2	C-C motif chemokine ligand 2	2.657
Cacna1a	calcium voltage-gated channel subunit alpha1 A	-5.096	Spp1	secreted phosphoprotein 1	10.971	Icam1	intercellular adhesion molecule 1	2.102
Cacng8	calcium voltage-gated channel auxiliary subunit gamma 8	-9.076	Col1a1	collagen, type I, alpha 1	15.053	Timp1	TIMP metallopeptidase inhibitor 1	6.297
Cacng7	calcium voltage-gated channel auxiliary subunit gamma 7	-4.678	Fos	FBJ osteosarcoma oncogene	-2.657	Cd44	CD44 molecule (Indian blood group)	6.132

Shank1	SH3 and multiple ankyrin repeat domains 1	-28.890	Tgfbr2	transforming growth factor, beta receptor 2	2.579	Spp1	secreted phosphoprotein 1	9.088
Rasgrf1	RAS protein-specific guanine nucleotide-releasing factor 1	-14.100	Sparc	secreted protein acidic and cysteine rich	2.122	Ccl19	C-C motif chemokine ligand 19	3.688
Cacnb1	calcium voltage-gated channel auxiliary subunit beta 1	-6.682	Tgfb1	transforming growth factor, beta 1	2.095	Pf4	platelet factor 4	3.404
Cacnb3	calcium voltage-gated channel auxiliary subunit beta 3	-4.156	Bmp7	bone morphogenetic protein 7	5.416	Itgb2	integrin subunit beta 2	2.361
Cacnb2	calcium voltage-gated channel auxiliary subunit beta 2	-2.618	C3	complement component 3	8.255	Il18	interleukin 18	2.361
Cacnb4	calcium voltage-gated channel auxiliary subunit beta 4	-4.463	Cyba	cytochrome b-245 alpha chain	4.201	Cxcl13	C-X-C motif chemokine ligand 13	2.306
**cellular response to organic cyclic compound (total 166 genes)**			**Nervous system development (total 90 genes)**			**Wound healing (total 28 genes)**		
gene_symbol	description	Fc	gene_symbol	description	Fc	gene_symbol	description	Fc
Casp3	caspase 3	2.403	Cd44	CD44 molecule (Indian blood group)	7.931	Timp1	TIMP metallopeptidase inhibitor 1	6.297
Stat3	signal transducer and activator of transcription 3	2.550	Igf1	insulin-like growth factor 1	2.352	Col1a1	collagen, type I, alpha 1	13.868
Igf1	insulin-like growth factor 1	52.188	Mmp2	matrix metallopeptidase 2	2.172	Tgfb1	transforming growth factor, beta 1	2.045
Myc	myelocytomatosis oncogene	5.493	Spp1	secreted phosphoprotein 1	10.971	Dcn	decorin	6.114
Bdnf	brain-derived neurotrophic factor	-3.076	Cxcl12	C-X-C motif chemokine ligand 12	2.135	Cd44	CD44 molecule (Indian blood group)	6.132
Ccl2	C-C motif chemokine ligand 2	22.318	Gfap	glial fibrillary acidic protein	8.649	Col3a1	collagen type III alpha 1 chain	13.728
Rhoa	ras homolog family member A	2.253	Col3a1	collagen type III alpha 1 chain	18.130	Igf2	insulin-like growth factor 2	28.159
Egr1	early growth response 1	-3.205	Fos	FBJ osteosarcoma oncogene	-2.657	Postn	periostin	-2.405
Icam1	intercellular adhesion molecule 1	6.542	Vim	vimentin	13.221	Vim	vimentin	10.175
Actb	actin, beta	2.246	Mmp14	matrix metallopeptidase 14	6.517	Pf4	platelet factor 4	3.404
