# Supplementary material for: Sequential Transcriptome Changes in the Penumbra after Ischemic Stroke
Source: Int J Mol Sci. 2019 Dec 16;20(24):6349. doi: 10.3390/ijms20246349 (PMC6940916; doi:10.3390/ijms20246349)
Supplement: Supplementary file 1 [file ijms-20-06349-s001.zip › Table S2.pdf]

DEGs of higher degrees in PPI analysis

1week post stroke

Gene Symbol	Description	Degrees	Fc
Dlg4	discs large MAGUK scaffold protein 4	93	-8.41
Bdnf	brain-derived neurotrophic factor	84	-3.08
Gria1	glutamate ionotropic receptor AMPA type subunit 1	81	-4.58
Rhoa	ras homolog family member A	76	2.25
Mapk8	mitogen-activated protein kinase 8	76	-2.18
Gng3	G protein subunit gamma 3	74	-5.48
Grin2a	glutamate ionotropic receptor NMDA type subunit 2A	73	-3.68
Gnb5	G protein subunit beta 5	72	-3
Ptprc	protein tyrosine phosphatase, receptor type, C	72	15.69
Tp53	tumor protein p53	70	2.75
Hras	Harvey rat sarcoma virus oncogene	70	-2.11
Itpkb	inositol-trisphosphate 3-kinase B	69	2.32
Gnai1	G protein subunit alpha i1	68	-2.84
Mapk11	mitogen-activated protein kinase 11	68	-5.74
Camk2a	calcium/calmodulin-dependent protein kinase II alpha	67	-139.81
Gnai2	G protein subunit alpha i2	66	2.31
Icam1	intercellular adhesion molecule 1	64	6.54
Ccl2	C-C motif chemokine ligand 2	64	22.32
Mmp9	matrix metallopeptidase 9	63	17.34
Grm5	glutamate metabotropic receptor 5	62	-3.18
Grin2b	glutamate ionotropic receptor NMDA type subunit 2B	62	-2.39
Casp3	caspase 3	62	2.4
Nras	neuroblastoma RAS viral oncogene homolog	61	2.29
Fos	FBJ osteosarcoma oncogene	61	-2.45
Grm3	glutamate metabotropic receptor 3	61	-5.52
Grm1	glutamate receptor, metabotropic 1	60	-2.47
Stat3	signal transducer and activator of transcription 3	59	2.55
Gria2	glutamate ionotropic receptor AMPA type subunit 2	59	-8.97
Tlr4	toll-like receptor 4	59	3.46
Igf1	insulin-like growth factor 1	59	52.19
Cxcl10	C-X-C motif chemokine ligand 10	56	3.82
Ccr5	chemokine (C-C motif) receptor 5	55	3.14
Syk	spleen associated tyrosine kinase	55	8.47
Cnr1	cannabinoid receptor 1 (brain)	55	-3.65
Ccr7	C-C motif chemokine receptor 7	54	3.14
Tlr2	toll-like receptor 2	54	8.67
Gria4	glutamate ionotropic receptor AMPA type subunit 4	53	-5.81
Cx3cr1	C-X3-C motif chemokine receptor 1	52	3.93
Snap25	synaptosomal-associated protein 25	52	-226.77
Gad1	glutamate decarboxylase 1	52	-3.54

4weeks post stroke

Gene Symbol	Description	Degrees	Fc
Cd44	CD44 molecule (Indian blood group)	17	7.93
C1qb	complement component 1, q subcomponent, B chain	15	2.88
Fcgr2b	Fc fragment of IgG, low affinity IIb, receptor	14	2.78
Spp1	secreted phosphoprotein 1	12	10.97
Cd74	CD74 molecule	12	10.83
C4a	complement component 4A (Rodgers blood group)	12	7.5
C1qa	complement component 1, q subcomponent, A chain	12	2.72
C3	complement component 3	10	8.25
Cd14	CD14 molecule	10	2.22
Itgb2	integrin subunit beta 2	10	3.42
RT1-CE5	RT1 class I, locus CE5	8	2.99
RT1-A2	RT1 class Ia, locus A2	8	2.62
Spi1	Spi-1 proto-oncogene	8	2.15
Col1a1	collagen, type I, alpha 1	8	15.05
Serping1	serpin family G member 1	8	21.82
Tgfb1	transforming growth factor, beta 1	7	2.09
C1s	complement component 1, s subcomponent	7	4.93
Cfh	complement factor H	6	3.38
Ctsk	cathepsin K	6	3.21
Lamb2	laminin subunit beta 2	6	2.02
RT1-M6-2	RT1 class I, locus M6, gene 2	6	-2.21
Itgb4	integrin subunit beta 4	6	10.08
Tnc	tenascin C	5	2.62
Tgfbr2	transforming growth factor, beta receptor 2	4	2.58
Mrc1	mannose receptor, C type 1	4	4.12
Trem2	triggering receptor expressed on myeloid cells 2	4	2.17
Col4a3	collagen type IV alpha 3 chain	4	-2.04
Col3a1	collagen type III alpha 1 chain	4	18.13
Cyba	cytochrome b-245 alpha chain	3	4.2
Cd4	Cd4 molecule	3	4.08
Fos	FBJ osteosarcoma oncogene	3	-2.66
Acp5	acid phosphatase 5, tartrate resistant	2	2.16
Lgmn	legumain	1	2.18

8weeks post stroke

Gene Symbol	Description	Degrees	Fc
Cd44	CD44 molecule (Indian blood group)	14	6.13
Fcgr2b	Fc fragment of IgG, low affinity IIb, receptor	13	2.12
C1qb	complement component 1, q subcomponent, B chain	13	2.39
Cd74	CD74 molecule	12	15.2
C1qc	complement component 1, q subcomponent, C chain	11	2.68
Cd14	CD14 molecule	10	2.58

C4a	complement component 4A (Rodgers blood group)	10	5.42
Spp1	secreted phosphoprotein 1	10	9.09
RT1-A2	RT1 class Ia, locus A2	9	3.53
Itgb2	integrin subunit beta 2	9	2.36
C1qa	complement component 1, q subcomponent, A chain	9	2.73
Serping1	serpin family G member 1	8	16.63
Icam1	intercellular adhesion molecule 1	8	2.1
B2m	beta-2 microglobulin	7	2.17
Itgb4	integrin subunit beta 4	7	10.95
Cd40	CD40 molecule	7	2.28
Mrc1	mannose receptor, C type 1	6	3.12
Cfh	complement factor H	6	2.61
Col6a2	collagen, type VI, alpha 2	6	2.16
Lamb2	laminin subunit beta 2	6	2.15
Col1a1	collagen, type I, alpha 1	6	13.87
Tapbp	TAP binding protein (tapasin)	4	2.01
Cd4	Cd4 molecule	4	2.38
Cyba	cytochrome b-245 alpha chain	4	4.4
Thbs2	thrombospondin 2	4	3.49
Tnc	tenascin C	4	2.07
Col3a1	collagen type III alpha 1 chain	4	13.73
Thbd	thrombomodulin	3	2.95
Tap2	transporter 2, ATP-binding cassette, sub-family B	2	2.09
Colec12	collectin sub-family member 12	1	5.27
F10	coagulation factor X	1	2.11
